# Short-Communication: Ingestion of a Nucleotide-Rich Mixed Meal Increases Serum Uric Acid Concentrations but Does Not Affect Postprandial Blood Glucose or Serum Insulin Responses in Young Adults

**DOI:** 10.3390/nu12041115

**Published:** 2020-04-17

**Authors:** Mariana O. C. Coelho, Alistair J. Monteyne, Ishara D. Kamalanathan, Vesna Najdanovic-Visak, Tim J. A. Finnigan, Francis B. Stephens, Benjamin T. Wall

**Affiliations:** 1 Department of Sport and Health Sciences, College of Life and Environmental Sciences, University of Exeter, Exeter EX1 2LU, UK; m.coelho@exeter.ac.uk (M.O.C.C.); a.monteyne@exeter.ac.uk (A.J.M.); f.b.stephens@exeter.ac.uk (F.B.S.); 2 UK Research and Innovation, Polaris House, Swindon SN2 1FL, UK; ishara.kamalanathan@ukri.org; 3 Chemical Engineering and Applied Chemistry (CEAC), Energy and Bioproducts Research Institute (EBRI), Aston University, Birmingham B4 7ET, UK; v.najdanovic@aston.ac.uk; 4 Marlow Foods Ltd., Stokesley TS9 7AB, UK; tim.finnigan@quornfoods.com

**Keywords:** nucleotides, serum uric acid, metabolic health, mycoprotein

## Abstract

Circulating uric acid concentrations have been linked to various metabolic diseases. Consumption of large boluses of nucleotides increases serum uric acid concentrations. We investigated the effect of a nucleotide-rich mixed meal on postprandial circulating uric acid, glucose, and insulin responses. Ten healthy adults participated in a randomised, controlled, double-blind, crossover trial in which they consumed a mixed-meal containing either nucleotide-depleted mycoprotein (L-NU) or high-nucleotide mycoprotein (H-NU) on two separate visits. Blood samples were collected in the postabsorptive state and throughout a 24 h postprandial period, and were used to determine circulating uric acid, glucose, and insulin concentrations. Mixed meal ingestion had divergent effects on serum uric acid concentrations across conditions (time x condition interaction; *P* < 0.001), with L-NU decreasing transiently (from 45 to 240 min postprandially) by ~7% (from 279 ± 16 to 257 ± 14 µmol·L^−1^) and H-NU resulting in a ~12% increase (from 284 ± 13 to 319 ± 12 µmol·L^−1^ after 210 min), remaining elevated for 12 h and returning to baseline concentrations after 24 h. There were no differences between conditions in blood glucose or serum insulin responses, nor in indices of insulin sensitivity. The ingestion of a nucleotide-rich mixed-meal increases serum uric acid concentrations for ~12 h, but does not influence postprandial blood glucose or serum insulin concentrations.

## 1. Introduction

Free ribonucleic acid (RNA) naturally occurs to varying degrees in many dietary protein sources. Upon ingestion, RNA is digested and absorbed as its constituent nitrogenous nucleotide bases (pyrimidines and purines), pentose sugars and phosphate ions. The primary end-product of purine metabolism is uric acid, which is mainly formed in the liver, transported to the kidneys and excreted in urine [1]. Human consumption of large boluses (>2 g/day) of isolated or (single-cell) yeast derived nucleotides has been shown to result in acute increases in circulating uric acid concentrations [2,3,4]. Epidemiological and observational data have reported that circulating uric acid concentrations positively correlate with development of gout [5], hypertension [6], and metabolic syndrome [7], and are a predictor of type 2 diabetes [8], though causation (particularly by diet) has not been established. Nevertheless, these findings informed the FAO/WHO/UNICEF (Food and Agriculture Organization/ World Health Organization/United Nations Children’s Fund). Protein Advisory Group recommendation to limit the additional dietary nucleic acid load from single cell protein novel foods to no more than 2 g/day [9,10]. How this translates to practical nutrition is unclear, since the ingestion of a high nucleotide containing mixed meal upon circulating uric acid concentrations has not been investigated. Moreover, whether acute elevations of serum uric acid influence, and therefore may be causatively linked to, markers of metabolic health, such as postprandial insulin sensitivity or glucose tolerance, is also unknown.

Mycoprotein is a single celled fungal-derived protein rich food source produced by the continuous cultivation of *Fusarium venenatum* and is naturally rich in RNA-derived nucleotides. To comply with the FAO/WHO/UNICEF Protein Advisory Group recommendations, industrial production of mycoprotein for commercial use involves the reduction of RNA content from ~10% to ~2% of its dry weight. This is achieved by an industrially expensive process of subjecting the culture broth to a short heat treatment (68 °C, 20 min) following fermentation, which allows for (endogenous) enzymatic breakdown of nucleotides, without affecting protein content [11]. We have previously shown that this process is effective, since the ingestion of nucleotide depleted mycoprotein up to quantities of 40 g did not influence postprandial circulating uric acid concentrations [12], with even 60 g causing only a transient (~2 h) and modest (from ~350 to ~380 µmol·L^−1^; well below clinically relevant thresholds of >420 µmol·L^−1^ [13]) rise, and in line with the consumption of other protein sources [14]. However, it is not known what impact the ingestion of non-nucleotide depleted mycoprotein has upon the postprandial serum uric acid response. By obtaining nucleotide depleted and non-depleted mycoprotein containing food products, we were able to test the hypothesis that the ingestion of a single nucleotide-rich mixed meal would acutely increase circulating uric acid concentrations and impair postprandial glucose handling.

## 2. Materials and Methods 

### 2.1. Participants and Medical Screening

Ten healthy young adults (age: 25 ± 1 years; BMI: 24.4 ± 1.0 kg/m^2^; male = 4 and female = 6) participated in the present study. Participants’ characteristics are presented in Table 1. Prior to participating, each subject attended a screening visit to ensure eligibility. Blood pressure, body mass, height, and body composition (determined by air displacement plethysmography: Bodpod; Life Measurement, Inc., Concord, CA, USA) were measured at screening. Smokers and participants taking regular medication or suffering from chronic diseases were excluded. Participants included were recreationally active (partook in regular exercise or sport at a non-competitive level, two to five days a week), normotensive, and had a BMI between 18.5 and 30 kg/m^2^. All participants were informed of the study’s purposes, procedures and risks, and provided written informed consent. The study was approved by the University of Exeter’s Sport and Health Sciences Ethics Committee (Ref No: 170712/A/01) in accordance with the Declaration of Helsinki, and was registered at ClinicalTrials.gov (NCT03568864) following the Consolidated Standards of Reporting Trials (CONSORT; Table S1 and Figure S1).

### 2.2. Experimental Protocol

The present study was a randomised, controlled, double-blind, crossover trial, where participants consumed a meal containing either a low (L-NU) or high (H-NU) amount of dietary nucleotides (1.96% and 8.83% of the mycoprotein (dry weight) in L-NU and H-NU, respectively; Table 2 details full nutritional composition of the test meals). 

Participants reported to the laboratory at ~08.00 a.m., after an overnight fast and refraining from intense exercise and alcohol consumption for at least 24 h, to undertake two identical experimental test days, separated by a two-week washout period. A cannula was placed retrogradely in a dorsal hand vein and the hand was then placed in a heated box (55 °C) for arterialised-venous blood sampling [15]. A fasted baseline arterialised-venous blood sample was collected and participants ingested the test meal, consisting of a sandwich made of three slices of bread (132 g; Kingsmill 50/50 medium, Allied Bakeries, UK), two packets of mayonnaise (28 g; Hellmann’s, Unilever, United States) and eight Quorn vegetarian chicken slices (100 g; Quorn, Marlow Foods, UK), containing either the nucleotide-depleted (L-NU) mycoprotein or the nucleotide-rich (H-NU) mycoprotein (77 g wet weight; corresponding to 19 g dry weight) in 10 min or less (with the exact time being recorded for each participant in the first visit and replicated on the second experimental test day). Arterialised-venous blood samples were then collected for a 4 h postprandial period, at 15 min intervals for the first hour and at 30 min intervals subsequently. Afterwards, participants were allowed to leave the laboratory and were instructed to return at 8, 12, and 24 h after the ingestion of the sandwich for further (venous) blood sample collection via venepuncture. Participants kept a diary detailing their food intake and physical activity during the 24 h period of the first experimental visit and replicated this for the second experimental visit.

### 2.3. Plasma and Serum Collection and Analyses

One mL of each blood sample was collected into FX blood collection tubes (Becton Dickinson, Franklin Lakes, NJ, USA) and glucose was immediately analysed using the YSI 2300 STAT PLUS Biochemistry Analyser (YSI, Yellow Springs, OH, USA). Four mL of each blood sample was collected into SST tubes (Becton Dickinson, Franklin Lakes, NJ, USA) and left at room temperature for at least 30 min to clot, before being centrifuged at 4 °C and 4000 RPM. The aliquoted serum samples were analysed for uric acid concentrations using the Roche Cobas 702 module of the Cobas 8000 analyser (Roche, Basel, Switzerland) and Roche Uric Acid Kits (Cobas; UA2) in the Clinical Chemistry department of the Royal Devon and Exeter NHS Foundation Trust. Serum insulin concentrations were analysed using DRG ELISA kits (DRG International, Springfield, NJ, USA). An adapted Cederholm insulin sensitivity index (Cederholm ISI; accounting for the carbohydrate intake from the mixed meal) was calculated using the blood glucose and serum insulin concentrations measured in the fasting state and during the 4 h post-prandial period, to provide an indirect measure of peripheral insulin sensitivity.

### 2.4. Statistical Analyses

All data are expressed as means ± standard errors (SEM). Serum uric acid, blood glucose, and serum insulin concentrations were analysed using two-way ANOVAs (with condition and time as factors). Bonferroni post hoc tests were performed in the event of significant main effects. Additionally, incremental Area Under the Curves (iAUCs) were calculated for blood glucose and serum insulin, and paired t-tests were performed to detect any significant effect of condition. The adapted Cederholm ISI data were also analysed using a paired t-test.

## 3. Results

### 3.1. Serum Uric Acid

**Figure 1:**
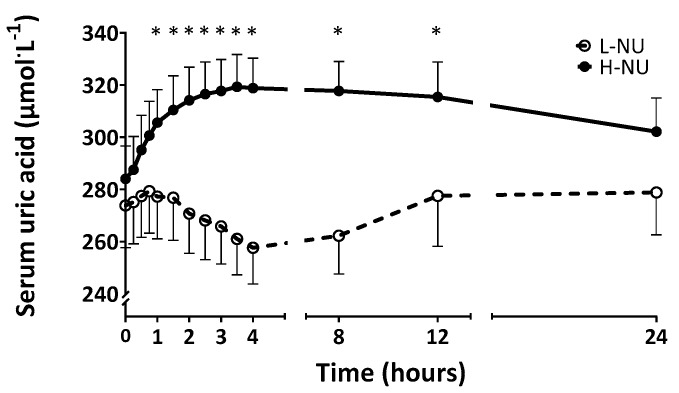
Temporal response of serum uric acid concentrations during a 24 h period following the consumption of a nucleotide-depleted (L-NU) or nucleotide-rich (H-NU) mycoprotein-based mixed meal in healthy young adults (*n* = 10). Data were analysed using a two-way ANOVA and Bonferroni post-hoc tests. Values are means with SEM represented by vertical bars. Main effect of time (*P* < 0.01), condition (*P* > 0.05) and time x condition interaction (*P* < 0.0001). * denotes significantly different from corresponding baseline value.

Serum uric acid concentrations during the 24 h experimental period are reported in Figure 1. Baseline fasting serum uric acid concentrations were not different between conditions (274 ± 16 µmol·L^−1^ in L-NU and 284 ± 13 µmol·L^−1^ in H-NU; *P* > 0.05). Meal ingestion influenced serum uric acid concentrations (effect of time; *P* < 0.001) and it did so divergently between groups (time x condition interaction; *P* < 0.001). In L-NU, meal ingestion modestly (~7 %) and transiently (from 45 min (279 ± 16 µmol·L^−1^) to 4 h (257 ± 14 µmol·L^−1^) post meal consumption) decreased serum uric acid concentrations, with baseline levels restored by 8 h post meal ingestion. Conversely, in the H-NU condition, following meal ingestion serum uric acid concentrations rose steadily by ~12 %, peaking after 210 min (319 ± 12 µmol·L^−1^; *P* < 0.05), remaining elevated for 12 h and returning to baseline concentrations only after 24 h (302 ± 13 µmol·L^−1^; *P* > 0.05).

When looking at sex based differences, postabsorptive serum uric acid concentrations were higher in male compared with female participants (314 ± 13 and 264 ± 14 µmol·L^−1^, respectively; *P* < 0.05). Meal ingestion influenced serum uric acid concentrations similarly across sexes and in line with the cohort as a whole (effect of time, *P* < 0.0001; trend for a time x condition interaction, *P* = 0.07) with similar absolute rises of ~30 to 40 µmol·L^−1^ (peaking after 210 min at 345 ± 11 µmol·L^−1^ for males and after ~8 h at 305 ± 14 µmol·L^−1^ for females), though this represented seemingly, but not significantly, differing rises expressed as a percentage across sexes (9 ± 2 and 16 ± 4 % for males and females, respectively; *P* > 0.05). In addition, females displayed more persistent elevations of serum uric acid concentrations with 24 h not being sufficient to return to baseline levels (after ~24 h: 292 ± 21 µmol·L^−1^).

### 3.2. Blood Glucose and Serum Insulin

**Figure 2:**
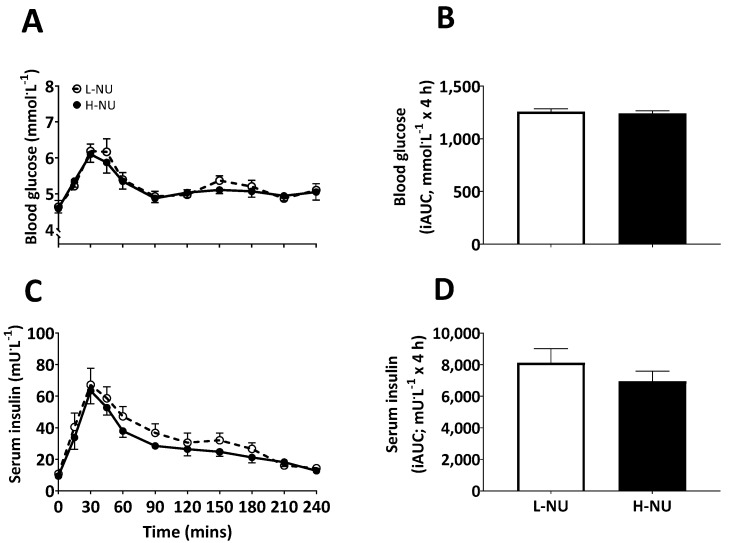
Temporal response and incremental area under the curve of blood glucose (**A**,**B**) and serum insulin (**C**,**D**) concentrations during the 4 h post-prandial period following the consumption of a nucleotide-depleted (L-NU) or nucleotide-rich (H-NU) mycoprotein-based mixed meal in healthy young adults (*n* = 10). Data were analysed using two-way ANOVAs. Incremental area under the curve (iAUC) data were analysed using paired t-tests. Values are means with SEM represented by vertical bars. Main effects of time, condition, time x condition interaction and paired t-tests (all *P* > 0.05).

Temporal blood glucose and serum insulin concentrations for the 4 h post prandial period are displayed in Figure 2A,C. Fasting blood glucose and serum insulin concentrations did not differ between conditions at baseline (both *P* > 0.05). For both measures, there was a significant effect of time (*P* < 0.0001), such that meal ingestion increased blood glucose and serum insulin concentrations (peaking at 30 min; glucose: 6.2 ± 0.2 and 6.1 ± 0.2 mmol·L^−1^, and insulin: 67 ± 10 and 63 ± 8 mU·L^−1^, for L-NU and H-NU, respectively) and to a similar degree for both conditions (condition and time x condition interaction; *P* > 0.05). Blood glucose iAUC and serum insulin iAUC during the OGTT (displayed in Figure 2B,D) were also not different between conditions (*P* > 0.05). Consequently, indirect peripheral insulin sensitivity calculated using the adapted Cederholm ISI did not differ between conditions (61 ± 3 and 64 ± 3 mg·L^2^·mmol^−1^·mU^−1^·min^−1^ for L-NU and H-NU, respectively; *P* > 0.05).

## 4. Discussion

We report that the consumption of a high-nucleotide mixed meal transiently (for 12 h) increased serum uric acid concentrations, but did not influence postprandial circulating glucose and insulin concentrations, or an adapted Cederholm insulin sensitivity index, compared with the ingestion of a low nucleotide control meal in healthy young adults. 

Higher circulating uric acid concentrations are associated with a variety of inflammatory and/or metabolic disorders, such as gout [5], hypertension [6], type 2 diabetes [8], and metabolic syndrome [7], leading to speculations of a causative role [16]. Dietary nucleotides have been implicated as the link between circulating uric acid and such disease progression, since daily short-term (5–9 days) ingestion of large quantities (>2 g) (isolated from yeast) has been shown to increase serum uric acid concentrations above clinically acceptable thresholds in healthy adults [2,3]. However, it is not clear whether this is the case within the context of a more nutritionally relevant mixed meal. Here, we report a high-nucleotide containing (i.e., 1.7 g) mixed meal increased serum uric acid concentrations within 1 h, and for a duration of 12 h post consumption (see Figure 1). This ~12 % rise (peaking at 319 µmol·L^−1^) was transient (returning to near basal levels the following day) and not close to clinically significant levels (i.e., ~420 µmol·L^−1^ in men, ~360 µmol·L^−1^ in pre-menopausal women [17]) in any of the participants. Interestingly, when subdividing our participants based on sex, some differences were evident. Namely, in accordance with the literature [17], males displayed higher postabsorptive serum uric acid concentrations compared with females, and although the absolute rise following the high nucleotide meal was similar between sexes, the higher relative rise amongst females translated to a more persistent elevation (i.e., at 24 h). The mechanism by which nucleotide rich meal ingestion elevates circulating uric acid concentrations likely relates to increased uric acid production as a metabolic end-product without any appreciable increase in (at least over the immediate postprandial period) urinary clearance. While we did not collect urine samples to confirm this, recent animal data report that experimental alterations in circulating uric acid concentrations are regulated at the level of renal handling [18]. However, this work also suggested that the direct mechanism was the level of circulating insulin, which is not consistent with our finding of equivalent postprandial insulin responses across conditions. Future (intervention) studies are warranted to assess whether repeated meals result in persistently and cumulatively (i.e., beyond clinically accepted levels) elevated serum uric acid concentrations, how this affects temporal rates of urinary uric acid clearance and to further probe the mechanisms underpinning nutritional uric acid balance in health and disease.

Making our current data more striking was the observation that serum uric acid concentrations decreased (from 45 min to 4 h post meal ingestion) under the control condition. This implies that the well documented protective effect of some dietary protein sources per se on circulating uric acid concentrations (shown previously with dairy and some plant based proteins; [19,20,21]), due to competitive tubular reabsorption and increased uric acid urinary clearance (either directly or indirectly via greater urea production), outweighed any impact of the low nucleotide content (i.e., 0.38 g) of this meal. Since vegan diets have been associated with higher circulating uric acid concentrations [21,22]—proposed to be attributable to the lack of dairy in the diet—and whilst we did not investigate this, our data imply nucleotide depleted mycoprotein may be a suitable protein choice to manage uric acid levels in these populations.

A potential mechanism by which circulating uric acid concentrations may be causative of declining health status is not clear. Numerous experiments performed in vitro [23,24,25,26,27,28] have shown that uric acid is a pro-oxidant in the intracellular environment, implying uric acid may contribute to metabolic dysfunction by promoting cellular oxidative stress [16]. Here we show that the increase in circulating uric acid concentrations following the nucleotide rich meal was not associated with any impairment in postprandial glucose handling or on an insulin sensitivity index. Though only addressing the acute response to bolus consumption, our data do not support that nutritionally induced acute increases in circulating uric acid concentrations impair markers of metabolic health. Intervention studies addressing the impact of habitual (rather than single meal) dietary nucleotide load on circulating uric acid concentrations and indices of metabolic health will be required to further elucidate the link between circulating uric acid, cellular oxidative stress, and metabolic health. 

## 5. Conclusions

In conclusion, the ingestion of a nucleotide-rich mixed meal increases serum uric acid concentrations for ~12 h, but does not influence postprandial blood glucose or serum insulin concentrations. 

## Figures and Tables

**Table 1:** Participants’ characteristics.

Sex	6 female/4 male
Age (years)	25 ± 1
Height (cm)	171 ± 3
Body mass (kg)	72 ± 4
Body mass index (kg/m^2^)	24.4 ± 1.0
Body fat (% of body mass)	22 ± 4
Lean mass (kg)	57 ± 5
Systolic blood pressure (mmHg)	117 ± 5
Diastolic blood pressure (mmHg)	68 ± 2

**Table 2:** Nutritional composition of the experimental meals.

	L-NU	H-NU
Energy and macronutrients		
Energy (kJ)	2519	2519
Energy (kcal)	602	602
Protein (g)	28	28
Carbohydrate (g)	58	58
Fat (g)	26	26
Nucleotides (in dry mycoprotein)		
CMP (g/%)	-	0.12/0.62
UMP (g/%)	0.05/0.26	0.08/0.44
GMP (g/%)	0.04/0.21	0.05/0.28
TMP (g/%)	0.09/0.46	0.57/3.00
CDP (g/%)	0.04/0.19	0.16/0.84
UDP (g/%)	0.01/0.07	0.06/0.34
CTP (g/%)	0.09/0.47	0.60/3.18
ADP (g/%)	0.00/0.02	0.02/0.08
TTP (g/%)	-	0.01/0.04
ITP (g/%)	0.06/0.29	-
ATP (g/%)	-	0.00/0.01
Total nucleotides (g/%)	0.38/1.96	1.70/8.83

Abbreviations: L-NU, Low nucleotide condition; H-NU, High nucleotide condition; CMP, Cytidine monophosphate; UMP, Uridine monophosphate; GMP, Guanosine monophosphate; TMP, Thymidine monophosphate; CDP, Cytidine diphosphate; UDP, Uridine diphosphate; CTP, Cytidine triphosphate; ADP, Adenosine diphosphate; TTP, Thymidine triphosphate; ITP, Inosine triphosphate; ATP, Adenosine triphosphate.

## Supplementary Materials

The following are available online at https://www.mdpi.com/2072-6643/12/4/1115/s1, Figure S1: CONSORT 2010 Flow Diagram, Table S1: CONSORT 2010 checklist of information to include when reporting a randomised trial *.
